# Circ‐PGAM1 promotes malignant progression of epithelial ovarian cancer through regulation of the miR‐542‐3p/CDC5L/PEAK1 pathway

**DOI:** 10.1002/cam4.2929

**Published:** 2020-03-13

**Authors:** Chunmei Zhang, Yang Li, Wancheng Zhao, Guipeng Liu, Qing Yang

**Affiliations:** ^1^ Department of Obstetrics and Gynecology Shengjing Hospital of China Medical University Shenyang China

**Keywords:** CDC5L, circ‐PGAM1, EOC, miR‐485‐5p, PEAK1

## Abstract

**Background:**

Epithelial ovarian cancer (EOC) is the most common ovarian malignant cancer. Circular RNA is a type of endogenous noncoding RNA and is considered as a novel regulatory molecule in the development and progression of tumors. This study investigated the expression and functions of a circular RNA, circular‐phosphoglycerate mutase 1 (circ‐PGAM1), in EOC tissues and cells.

**Methods:**

The expression of circ‐PGAM1 and miR‐542‐3p in EOC was analyzed using quantitative RT‐PCR. Immunohistochemistry and western blot were performed to confirm the localization and expression of cell division cycle 5‐like (CDC5L) and pseudopodium enriched atypical kinase 1 (PEAK1) in EOC tissues. Cell lines (CAOV3 and OVCAR3) overexpressing or silencingcirc‐PGAM1 and miR‐542‐3p were established to explore the functions of circ‐PGAM1 and miR‐542‐3p in ovarian cancer cells. Furthermore, dual‐luciferase reporter assay was performed to study the interactions between circ‐PGAM1 and miR‐542‐3p and between miR‐542‐3p and CDC5L. CCK‐8, transwell, and flow cytometry were used to study the effect of circ‐PGAM1 and miR‐542‐3p on cell biological behaviors including proliferation, migration, invasion, and apoptosis. The interaction between CDC5L and the *PEAK1* gene promoter was confirmed using chromatin immunoprecipitation (ChIP).

**Results:**

Circ‐PGAM1 was upregulated in EOC tissues, whereas linear PGAM1 was not deregulated in EOC tissues. Silencing of circ‐PAGM1 inhibited proliferation, migration, and invasion of ovarian cancer cells and promoted cell apoptosis. MiR‐542‐3p was downregulated in EOC tissues, and miR‐542‐3p overexpression inhibited malignant progression of ovarian cancer cells. Circ‐PGAM1 directly interacted with miR‐542‐3p, with mutual negative feedback between them. CDC5L was a direct target of miR‐542‐3p and played an oncogenic role in ovarian cancer cells. Furthermore, the CDC5L protein binds directly to the PEAK1 promoter to promote its transcription. PEAK1 overexpression activated ERK1/2 and JAK2 signaling pathways and promoted malignant biological behaviors of ovarian cancer cells. Circ‐PAGM1 silencing combined with miR‐542‐3p overexpression played the greatest anticancer role in vivo.

**Conclusion:**

The circ‐PGAM1/miR‐542‐3p/CDC5L/PEAK1 pathway played an important role in the progression of ovarian cancer and might be a novel therapeutic target for ovarian cancer.

## Graphical abstract

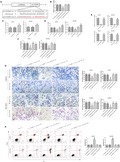

## 1 INTRODUCTION

Among the three major malignant tumors of the female reproductive system, ovarian cancer has the highest mortality. Epithelial ovarian cancer (EOC) is the most common pathological type of ovarian cancer and accounts for approximately 90% of the incidence of ovarian cancer.^1^, ^2^ EOC can be classified into type I and type II EOC based on clinicopathological parameters.^3^ Although there was improvement in medical treatment in the past 40 years, the 5‐year survival rate of EOC is still lower than 50%. For EOC at FIGO stages III and IV, the reported 5‐year survival rates are only 41% and 20%, respectively.^1^ The major reasons for this high mortality are the lack of effective early diagnosis strategies and recurrence resulting from chemotherapy resistance. EOC, especially type II EOC, is mostly accompanied by hereditary mutations, which in turn cause activation of a series of oncogenes and inactivation of tumor suppressor genes. The resulting signaling pathway abnormalities lead to EOC development.^4^ Therefore, identifying key genes and regulatory pathways in the development and progression of EOC could promote early diagnosis of ovarian cancer and improve treatment strategies to increase the survival of ovarian cancer patients.

Circular RNAs (circRNAs) are a type of special endogenous noncoding RNAs. In contrast to traditional linear RNAs, circRNAs do not have a 5′‐end cap and a 3′‐end poly(A) tail. CircRNAs form a closed circular structure using covalent bonds through reverse splicing; therefore, they are not affected by RNA exonucleases and are more stably expressed.^5^ There are many types of circRNAs, which are extensively present in eukaryotic cells. Most circRNAs are composed of exons and are located in the cytoplasm.^6^ Studies in recent years^7^, ^8^, ^9^, ^10^ have shown that circRNAs have complex functions, including acting as competing endogenous RNAs (ceRNAs) to exert microRNA (miRNA) sponge functions, regulating gene transcription and translation, and interacting with proteins. Some circRNAs even encode proteins. At present, their function as miRNA sponge is being widely studied. Through interactions with tumor‐associated miRNAs, circRNAs play important regulatory roles in various types of tumors.^11^, ^12^, ^13^, ^14^

Circ‐PGAM1 is transcribed from its parental gene *phosphoglycerate mutase 1 (PGAM1)*. The PGAM1 protein is one of the key enzymes in the glycolysis pathway, which promotes glucose metabolism and energy production through catalyzing the conversion of 3‐phosphoglycerate (3‐PG) to 2‐phosphoglycerate (2‐PG).^15^ PGAM1 influences other metabolic pathways by regulating the balance between 3‐PG and 2‐PG conversion in the biosynthesis and metabolism and maintaining the redox steady state in cells. PGAM1 is usually upregulated in TP53‐deficient tumors and promotes tumor growth by coordinating glycolysis and biosynthetic metabolism.^16^ PGAM1 can promote proliferation and metastasis of tumors cells in malignant tumors such as small cell lung cancer, pancreatic ductal adenocarcinoma, and oral squamous cell carcinoma by playing a tumor‐promoting role.^17^, ^18^, ^19^ However, there have been no reports on the function of circ‐PGAM1 in cancers. Our preliminary study showed that circ‐PGAM1 (hsa_circ_0019340) is highly expressed in ovarian cancer tissues. Therefore, we hypothesized that circ‐PGAM1 might have a tumor‐promotion function similar to that of its parental gene PGAM1 in the development and progression of ovarian cancer.

MicroRNAs (miRNAs) are another type of noncoding RNAs. They bind to the 3′ untranslated region (UTR) of mRNAs to cause mRNA degradation or inhibit their translation; therefore, they can regulate gene expression posttranscriptionally.^20^ According to the different functions of their regulated target genes, miRNAs can act as an oncogene or tumor suppressor gene to exert important functions in the development and progression of malignant tumors.^21^, ^22^ MiR‐542‐3p can promote P53 expression by activating P53 targets and binding to oncogenes such as Survivin to inhibit cell proliferation, migration, and invasion, promote cell apoptosis and play a tumor‐suppressing role in various types of tumors.^23^, ^24^, ^25^, ^26^ Analysis using Starbase v2.0 bioinformatics software predicted a binding site between circ‐PGAM1 and miR‐542‐3p. According to the ceRNA hypothesis, we speculated that circ‐PGAM1 could competitively bind to miR‐542‐3p and attenuate the inhibitory function of miR‐542‐3p on target genes to upregulate target gene expression and further regulate the malignant biological behaviors of ovarian cancer cells. The mechanism underlying the interaction of circ‐PGAM1/miR‐542‐3p in ovarian cancer has not been reported.

The CDC5L (cell division cycle 5‐like) protein is a mammalian homologue of Cdc5 first discovered in fission yeasts that is extensively expressed in mammals. It is a core component of the PRP19‐CDC5L complex and participates in the regulation of premRNA splicing.^27^ CDC5L can also act as a transcription factor to activate downstream target genes.^28^, ^29^, ^30^, ^31^ A genome‐wide gene expression analysis revealed that CDC5L regulates the expression of a series of genes in mitosis and DNA damage repair and plays important roles in normal development of various types of tissues and organs in human.^32^ CDC5L is highly expressed in malignant tumors such as liver cancer, colorectal cancer, and prostate cancer and plays a tumor‐promoting role.^28^, ^33^, ^34^ TargetScan predicted that miR‐542‐3p has a binding site in the 3′UTR of the CDC5L mRNA.

PEAK1 (pseudopodium enriched atypical kinase 1) was first discovered in cellular pseudopodia and named by Wang et al.^35^ It is a nonreceptor tyrosine kinase with catalytic activities and belongs to the NKF3 family. PEAK1 is expressed in many types of tissues and cells. PEAK1 contains binding sites or substrates for cytoskeletal effector proteins such as Src, ERK, Crk, and Shc and can interact with them to influence their phosphorylation levels; therefore, PEAK1 plays an important role in cytoskeletal regulation and cell migration. It is also involved in the malignant progression of tumor cells. PEAK1 has currently been confirmed to promote cell proliferation and migration, inhibit cell apoptosis, and play a tumor‐promoting role through ERK1/1 and JAK2 pathways in breast cancer, colorectal cancer, and lung cancer.^35^, ^36^, ^37^ However, relevant functions in ovarian cancer have not been reported. By the bioinformatics database GTRD (Gene Transcription Regulation Database), PEAK1 was predicted to be a regulated target of CDC5L, and by another database JASPAR, CDC5L protein was predicted to have a binding site in the promoter region of *PEAK1* gene. Therefore, it was hypothesized that CDC5L might bind to the PEAK1 promoter to regulate PEAK1 expression and further activate downstream signaling pathways to synergistically play a tumor‐promoting role.

This study detected the expression levels of circ‐PGAM1, miR‐542‐3p, CDC5L, and PEAK1 in ovarian cancer tissues, validated the interaction between circ‐PGAM1 and miR‐542‐3p, between miR‐542‐3p and CDC5L, and between CDC5L and PEAK1, investigated their regulatory functions in the malignant biological behaviors of ovarian cancer, and elucidated the underlying mechanisms to uncover novel targets for ovarian cancer treatment.

## 2 MATERIALS AND METHODS

### 2.1 Clinical specimens

Primary EOC tissues and normal ovary tissues were collected from the patients undergoing surgical excision at the Department of Obstetrics and Gynecology, Shengjing Hospital of China Medical University. No patient received radiotherapy, chemotherapy, or hormone therapy before surgery. Primary EOC tissues were divided into two groups: Type I group (n = 15) and Type II group (n = 15). Type I group included Mucinous carcinoma, clear cell carcinoma, and low‐grade serous and endometrioid carcinoma. Type II group included high‐grade serous and endometrioid carcinoma and undifferentiated carcinoma. Normal ovarian tissues were used as the control group (n = 15). The histopathology types of the EOC tissues were confirmed independently by two pathologists.

### 2.2 Cell culture

The human ovarian cancer cell lines (CAOV3, SKOV3, OVCAR3, and ES‐2) and human embryonic kidney (HEK) 293T cells were purchased from the Institute of Biochemistry and Cell Biology, Chinese Academy of Sciences (Shanghai, China). CAOV3, SKOV3, OVCAR3, and ES‐2 cells were cultured routinely in RPMI 1640 culture medium containing 10% fetal bovine serum (FBS, Biological Industries). HEK‐293 cells were cultured in Dulbecco's modified Eagle medium (DMEM)/high glucose (GIBCO, USA) with 10% FBS. All cells were maintained in a humidified incubator at 37°C with 5% CO2 and tested to be mycoplasma‐free.

### 2.3 Fluorescence in situ hybridization (FISH)

For identification of circ‐PGAM1 and miR‐542‐3p localization in ovarian cancer cells, circ‐PGAM1 probe (Red‐labeled, China) (5′‐TGCATACCTGCGATCTATTGCACATCACTC‐3′) and miR‐542‐3p probe (green‐labeled, BersinBio, China) (5′‐TTTCAGTTATCAATCTGTCACA‐3′) were used. In brief, cells grown on coverslips were fixed with 4% paraformaldehyde at room temperature for 20 minutes, washed twice with 0.1% diethylpyrocarbonate solution and treated with 0.5% Triton X‐100 at room temperature for 10 minutes. The samples were dehydrated in a graded series of alcohol and air‐dried. The hybridization mix was prepared with circRNA probe and miRNA probe in hybridization solution. After the hybridization mix was added, the samples were denatured at 73°C for 5 minutes and hybridized in a humid and dark environment at 37°C for 16‐20 hours. The samples were washed sequentially with a preheated (42°C) solution consisting of 25% formamide and 2× saline sodium citrate (SSC), a preheated (39°C) solution consisting of 0.1%Nonidet P 40 and 2× SSC, 0.5 × SSC (39°C) and 0.2 × SSC (39°C). After being counterstained with 4′, 6‐diamidino‐2‐phenylindole for 10 minutes, the samples were mounted with fluorescence mounting medium and imaged with a fluorescence microscope (Carl Zeiss).

### 2.4 Cell transfections

Short‐hairpin circ‐PGAM1 (circ‐PGAM1 (−)) and PGAM1 (PGAM1 (−)) plasmids and their respective nontargeting sequence (negative control, NC); miR‐542‐3p agomir (pre‐miR‐542‐3p), miR‐542‐3p antagomir (anti‐miR‐542‐3p), and their respective nontargeting sequence (pre‐NC and anti‐NC) were synthesized (GenePharma). CDC5L full‐length (with 3′‐UTR) (CDC5L (+) or CDC5L) plasmid, short‐hairpin CDC5L (CDC5L (−)) plasmid, CDC5L (without 3′‐UTR) (CDC5L (non‐3′UTR)) plasmid, and their respective nontargeting sequence (CDC5L (+)‐NC or CDC5L (−)‐NC), PEAK1 full‐length (PEAK1 (+)) plasmid, short‐hairpin PEAK1 (PEAK1 (−)) plasmid, and their respective nontargeting sequence (PEAK1 (+)‐NC or PEAK1 (−)‐NC) were constructed (Life Technology). Cells were transfected in 24‐well plates using Lipofectamine 3000 reagent (Invitrogen) according to the manufacturer's instructions. Geneticin (G418; Invitrogen) was used to select stably transfected cells. Overexpressing and the silencing efficiency were evaluated by qRT‐PCR. To explore the effect of circ‐PGAM1 on ovarian cancer, cells were divided into three groups: Control group, circ‐PGAM1 (−)‐NC group, and circ‐PGAM1 (−) group. Similarly, to investigate the effect of miR‐542‐3p on ovarian cancer, cells were divided into five groups: Control group, pre‐NC group, pre‐miR‐542‐3p group, anti‐NC group, and anti‐miR‐542‐3p group. To investigate the effect of CDC5L on ovarian cancer, cells were divide into five groups: Control group, CDC5L (+)‐NC group, CDC5L (+) group, CDC5L (−)‐NC group, and CDC5L (−) group. In addition, to investigate the effect of PEAK1 on ovarian cancer, cells were divided into five groups: Control group, PEAK1 (+)‐NC group, PEAK1 (+) group, PEAK1 (−)‐NC group, and PEAK1 (−) group. Furthermore, to explore the underlying mechanism of circ‐PGAM1 regulating the malignant progression of ovarian cancer cells via impairing miR‐542‐3p, cells were divided into five groups: Control group, circ‐PGAM1(−)‐NC + pre‐NC group, circ‐PGAM1 (−) + miR‐542‐3p (+) group, circ‐PGAM1(−)‐NC + anti‐NC group, and circ‐PGAM1 (−) + anti‐miR‐542‐3p group. Furthermore, to determine the effect of miR‐542‐3p inhibiting the malignant progression of ovarian cancer cells via targeting CDC5L 3′‐UTR, cells were dived into four groups: miR‐542‐3p‐NC + CDC5L‐NC group, miR‐542‐3p + CDC5L‐NC group, miR‐542‐3p + CDC5L group, and miR‐542‐3p + CDC5L(non‐3′UTR) group.

### 2.5 RNA extraction, reverse transcription, and real‐time quantitative PCR (qRT‐PCR)

Total RNA was isolated from tissues and cells using the RNAiso Plus reagent (Takara Bio). RNA concentration and quality were detected at an absorbance of 260/280 nm using a Nanodrop Spectrophotometer (ND‐100, Thermo). One‐Step SYBR PrimeScript RT‐PCR Kit (Perfect Real Time) (RR066A, Takara Bio) was used for measurement of circ‐PGAM1 and mRNAs of PGAM1, CDC5L, and PEAK1. GAPDH was used as the endogenous control. In addition, RNase‐R was used to confirm the existent of circ‐PGAM1 and to eliminate the influence of liner RNAs. TaqMan miRNA Reverse Transcription Kit (Applied Biosystems) was used to generate cDNA from miRNA. qRT‐PCR was performed by using TaqMan Universal Master Mix II (Applied Biosystems) and Taq‐Man microRNA assays of miR‐542‐3p and U6 (Applied Biosystems). All kits were used according to the manufacturer's instructions. PCR reactions were performed in ABI 7500 Fast (Life Technologies) in triplicates and validated by the presence of one single peak in the melt curve. Gene expression relative to endogenous controls was calculated by 2 − ΔΔCt method.

### 2.6 Western blot

Total protein was extracted from tissues and cells using RIPA buffer supplemented with protease inhibitors (Beyotime) on ice, underwent SDS‐PAGE electrophoresis and then transferred to PVDF membrane (Millipore). After nonspecific bindings were blocked by 5% fat‐free milk at room temperature for 2 hours, membranes were incubated overnight at 4°C using primary antibodies as shown below: CDC5L (1:1000; Proteintech), PEAK1 (1:1000; Abcam), p‐ERK (1:1000; Cell Signaling), ERK (1:1000; Cell Signaling), p‐JAK1 (1:1000; Cell Signaling), JAK1 (1:1000; Cell Signaling), p‐JAK2 (1:1000; Cell Signaling), JAK2(1:1000; Cell Signaling), and GAPDH (1:2000; Proteintech). Then, the membranes were washed and incubated for 2 hours at room temperature with secondary antibodies (Proteintech) respectively. Immunoblots were visualized by ECL chemiluminescence detection system (Thermo Scientific) and the relative integrated density values (IDVs) were calculated based on GAPDH as the internal control.

### 2.7 Cell proliferation assay

CAOV3 and OVCAR3 cells were plated in 96‐well plates at a density of 2 × 103 cells per well. After the cells were transfected for 72 hours, 10 μL of Cell Counting Kit‐8 solution (CCK‐8, Dojin, Japan) was added into each well and the cells were incubated for 2 hours at 37°C. The absorbance at 450 nm was detected using a universal microplate reader.

### 2.8 Migration and invasion assays

CAOV3 and OVCAR3cells were resuspended in 200 μL serum‐free culture medium at a density of 5 × 104/mL and seeded in the upper chamber for cell migration assay (or precoated with 80 μL Matrigel solution [BD, USA] for cell invasion assay). Culture medium (600 µL) containing 10% FBS was added to the lower chambers. After incubation for 24 hours, the cells on the upper membrane surface were removed with cotton swabs carefully. The cells which had migrated or invaded to the lower side of the membrane were fixed using methanol/glacial acetic acid mixture and stained with 10% Giemsa (Dinguo, China). Five random fields were chosen to count cells for statistics under a microscope and photographs were taken.

### 2.9 Apoptosis analysis

Cellular apoptosis was detected by flow cytometry using the Annexin V‐phycoerythrin (PE)/7‐actinomycin D (7AAD) staining (BD, USA) according to the manufacturer's instructions. In brief, cells were trypsinized, washed with ice‐cold PBS, and stained with Annexin V‐APC/7‐AAD. After a 15‐min incubation, cell samples were analyzed using a flow cytometer (FACScan, BD, USA) and apoptotic fractions were analyzed by FlowJo software.

### 2.10 Dual‐Luciferase reporter assay

The circ‐PGAM1 and CDC5L 3′‐UTR binding sites to miR‐542‐3p were predicted by Starbase v2.0 (http://starbase.sysu.edu.cn/) and TargetScan (http://www.targetscan.org/), respectively. Then the sequence of circ‐PGAM1 containing the putative miR‐542‐3p binding sites was cloned into a pmirGLO Dual‐Luciferase Vector (Promega) to construct the reporter vector, circ‐PGAM1‐wild‐type (circ‐PGAM1‐Wt) (GenePharma). Similarly, the corresponding mutant of putative miR‐542‐3p binding site was used to construct the reporter vector, circ‐PGAM1‐mutated‐type (circ‐PGAM1‐Mut) (GenePharma). The 3′‐UTR sequence of CDC5L gene and its mutant of the putative miR‐542‐3p binding site were cloned into a pmirGLO Dual‐Luciferase Vector to construct the reporter vectors, CDC5L‐3′‐UTR‐wild‐type (CDC5L‐3′‐UTR‐Wt) and CDC5L‐3′‐UTR‐mutated‐type (CDC5L‐3′‐UTR‐Mut) (GenePharma), respectively. The pmirGLO vector (wild type sequence or mutant type sequence) and miR‐542‐3p agomir (or agomir NC) were transfected into HEK 293 T cells using Lipofectamine 3000. Relative luciferase activities were detected 48 hours after transfection by the Dual‐Luciferase Reporter Assay System (Promega).

### 2.11 RIP assay

RIP (RNA‐binding protein immunoprecipitation) assay was performed using EZ‐Magna RIP kit (Millipore) according to the manufacturer's protocol. Whole cell lysate of the circ‐PGAM1 groups and miR‐542‐3p groups was incubated with RIP immunoprecipitation buffer containing magnetic beads conjugated with human anti‐Argonaute2 (Ago2) antibody (Millipore) and negative control normal mouse IgG (Millipore). Samples were incubated with Proteinase K buffer and then the immunoprecipitated RNA was isolated. The purified RNA was then extracted and analyzed by qRT‐PCR to verify the presence of the binding targets using respective aforementioned primers.

### 2.12 ChIP assay

The ChIP assay was performed using Simple ChIP Enzymatic Chromatin IP Kit (Cell Signaling Technology) according to the manufacturer's protocol. The cells (CAOV3 and OVCAR3) were cross‐linked with 1% formaldehyde in culture medium and collected in lysis buffer. About 2% of the lysates were used as input control, and the other lysates were incubated with normal rabbit IgG or CDC5L antibody. Immunoprecipitated DNA was amplified by PCR with the following primers: PCR 1 (putative binding site) (F) 5′‐GCTTACTGACCTGCTGACGA‐3′, (R) 5′‐AGACGACGAGTGCGTGAAG‐3′; PCR 2 (control) (F) 5′‐TCGGGCAGTCTACATGTTCCTA‐3′, (R) 5′‐TTTGAAAGACCACACGATTGCT‐3′.

### 2.13 Tumor xenografts in nude mice

The pLenti6.3/V5eDEST Gateway Vector Kit (Life Technologies) was used to generate Lentivirus encoding miR‐542‐3p and its nontargeting sequence (negative control, NC). The miR‐542‐3p sequence was ligated to the pLenti6.3/V5eDEST vector, and the short‐hairpin RNA targeting human circ‐PGAM1 was ligated to the LV3‐CMV‐GFP‐Puro vector (GenePharma). Then pLenti6.3/V5eDEST‐miR‐542‐3p and LV3‐CMV‐GFP‐Puro‐circ‐PGAM1 vectors were generated. The ViraPower Packaging Mix was used to generate Lentivirus in 293FT cells. After infection, the CAOV3 and OVCAR3 cells stably expressing miR‐542‐3p (miR‐542‐3p(+)) and circ‐PGAM1 (circ‐PGAM1(−)) were obtained. The lentiviruses of miR‐542‐3p(+) were transduced in circ‐PGAM1(−) stably transfected cells to generate circ‐PGAM1(−) + miR‐542‐3p(+) cells. The nude mice were divided into five groups: control group, circ‐PGAM1(−)‐NC + miR‐542‐3p(+)‐NC group, circ‐PGAM1(−) group, miR‐542‐3p(+) group, and circ‐PGAM1 (−) + miR‐542‐3p(+) group. Each mouse was subcutaneously injected with 5 × 10^5^ cells in the right armpit. When a tumor was palpable, the tumor size was measured every 3 days, and its volume was calculated according to the formula: volume (mm^3^) = length × width^2^/2. Then all the mice were sacrificed 33 days after injection and the tumors were resected for further experiments. This part of the study involving animals was in accordance with Guidelines for Animal Care and Use of the authors' institution and was approved by the Institutional Animal Care and Use Committee of the authors' institution.

### 2.14 Statistical analysis

Data are presented as mean + standard deviation (SD). All statistical analyses were evaluated by SPSS 18.0 statistical software with the unpaired t test. Differences were considered to be significant when *P* < .05 (two‐sided). Corresponding significance levels were indicated in the figures.

## 3 RESULTS

### 3.1 Circ‐PGAM1 silencing inhibits proliferation, migration, and invasion and promotes apoptosis of ovarian cancer cells

**Figure 1:**
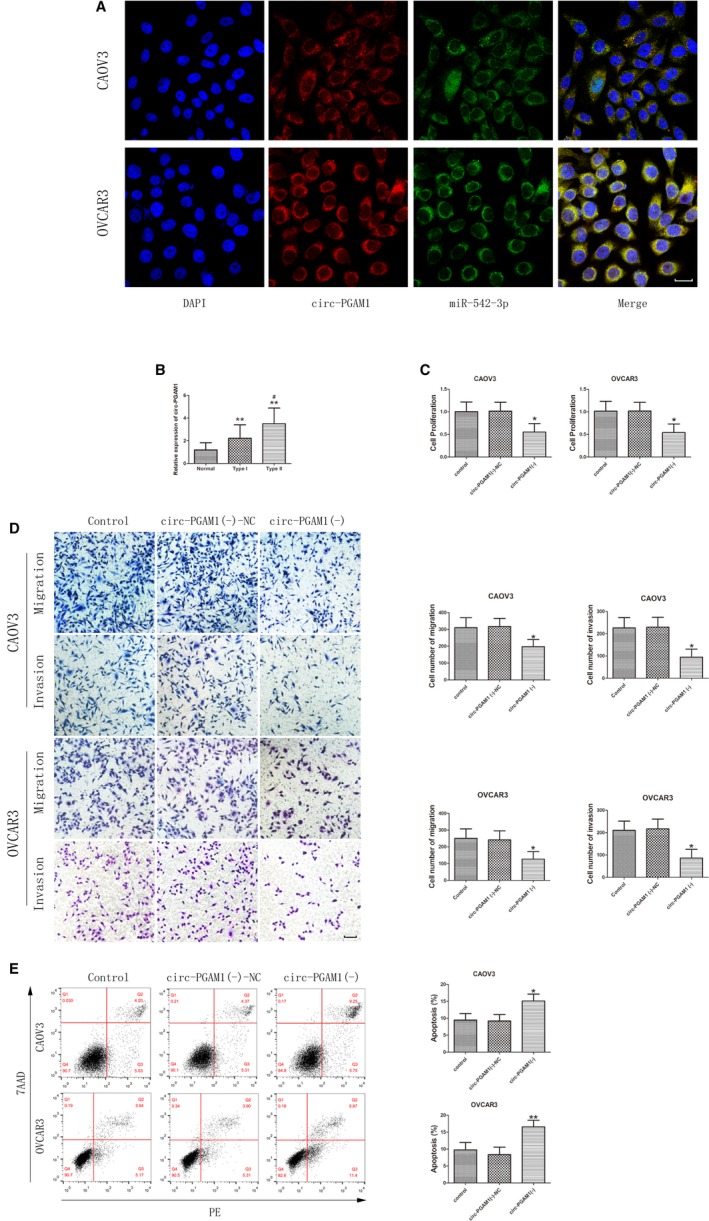
Circ‐PGAM1 functioned as an oncogene in epithelial ovarian cancer (EOC) tissues and cell lines. A, Fluorescence in situ hybridization (FISH) was used to determine the location of circ‐PGAM1 and miR‐542‐3p in CAOV3 and OVCAR3 (blue, DAPI nuclear staining; red, circ‐PGAM1; green, miR‐542‐3p). Scale bars represent 20 μm. B, Expression of circ‐PGAM1 in EOC tissue s of different types and normal ovarian tissues (data are presented as mean ± SD [n = 15, each group]; **P < .01 vs normal group; ^#^ *P* < .05 vs type I group). C, CCK‐8 assay was conducted to explore the effect of circ‐PGAM1 on proliferation of CAOV3 and OVCAR3 (data are presented as mean ± SD [n = 3, each group]; *P < .05 vs circ‐PGAM1 (−)‐NC group). D, Transwell assay was used to determine the effect of circ‐PGAM1on migration and invasion of CAOV3 and OVCAR3. Typical images and accompanying statistical plots were presented (data are presented as mean ± SD [n = 3, each group]; *P < .05 vs circ‐ PGAM1 (−)‐NC group). Photographs were taken at 200× magnification. Scale bar represents 100 μm. E, Flow cytometry was used to detect apoptosis of CAOV3 and OVCAR3with knockdown of circ‐PGAM1 (data are presented as mean ± SD [n = 3, each group]; *P < .05 vs circ‐ PGAM1 (−)‐NC group; **P < .01 vs circ‐ PGAM1 (−)‐NC group)

Fluorescence in situ hybridization (FISH) results showed that circ‐PGAM1 was mainly expressed in the cytoplasm (Figure 1A). To clarify the expression of circ‐PGAM1 in ovarian cancer tissues, the expression levels of circ‐PGAM1 in normal ovarian tissue, type I EOC, and type II EOC were detected using qRT‐PCR. The results showed that the expression level of circ‐PGAM1 in EOC tissues was significantly higher than in normal ovarian tissue and that the expression level in type II EOC was significantly higher than in type I EOC (Figure 1B). However, the expression levels of linear PGAM1 did not differ significantly between EOC tissues and normal ovarian tissues (Figure S1A). In addition, the detected RNA was validated as circRNA by adding ribonuclease R (RNase R). The results showed that circ‐PGAM1 expression was not affected by RNase R, whereas the expression level of linear PGAM1 was significantly reduced after treatment with RNase R (Figure S1B).

To further study the effect of circ‐PGAM1 on biological behaviors of ovarian cancer cells, the expression of circ‐PGAM1 in the ovarian cancer cell lines SKOV3, CAOV3, OVCAR3, and ES‐2was detected. As shown in Figure S1C, the expression levels of circ‐PGAM1 were higher in CAOV3 and OVCAR3. Therefore, CAOV3 and OVCAR3 were selected as the cell lines for investigation. Circ‐PGAM1 shRNA was transfected into cells to silence circ‐PGAM1. The transfection efficiency is shown in Figure S1D. PGAM1 expression was detected after circ‐PGAM1 silencing to confirm whether the circular form rather than the linear form of PGAM1 was inhibited. As shown in Figure S1E, the expression levels of PGAM1 did not differ significantly between the circ‐PGAM1(−) group and the circ‐PGAM1(−)‐NC group. In addition, PGAM1 was silenced to determine whether PGAM1(−) affects circ‐PGAM1 expression. The transfection efficiency of PGAM1 is shown in Figure S1F. The expression levels of circ‐PGAM1 did not differ significantly between the PGAM1(−) group and the PGAM1(−)‐NC group (Figure S1G). Furthermore, CCK‐8 assay showed that the proliferation rate of cells significantly decreased in the circ‐PGAM1(−) group compared to the circ‐PGAM1(−)‐NC group (Figure 1C). Transwell assay showed that the number of migrating and invading cells significantly decreased in the circ‐PGAM1(−) group compared to the circ‐PGAM1(−)‐NC group (Figure 1D). Flow cytometry showed that the apoptosis rate of cells significantly increased in circ‐PGAM1(−) group compared to circ‐PGAM1(−)‐NC group (Figure 1E). The above results indicate that circ‐PGAM1 silencing can inhibit the proliferation, migration, and invasion of ovarian cancer cells and promote apoptosis of ovarian cancer cells.

### 3.2 MiR‐542‐3p plays a tumor‐suppressing role in ovarian cancer cells

**Figure 2:**
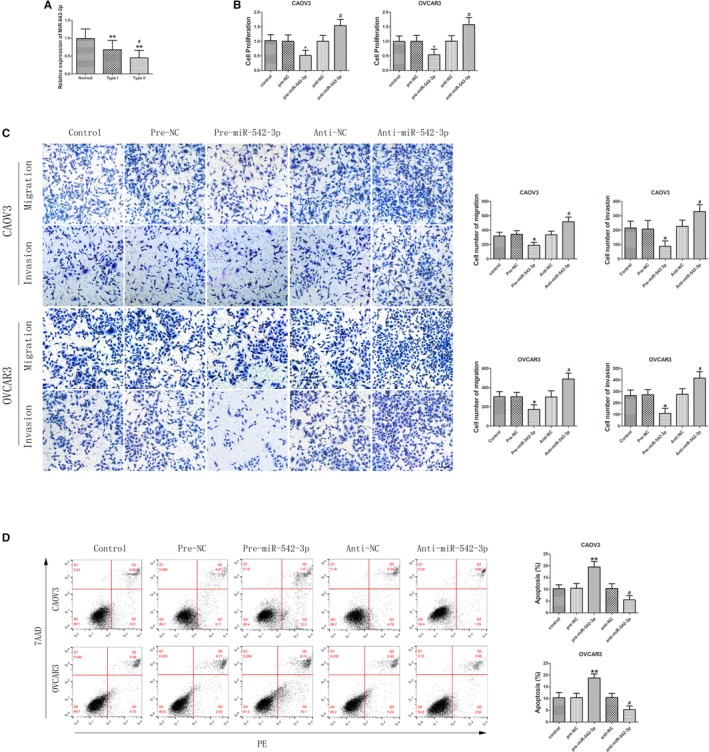
MiR‐542‐3p played an anti‐oncogenic role in epithelial ovarian cancer (EOC) tissues and cell lines. A, Expression of miR‐542‐3p in EOC tissues of different types and normal ovarian tissues (data are presented as mean ± SD [n = 15, each group]; **P < .01 vs normal group; ^#^ *P* < .05 vs type I group). B, Effect of miR‐542‐3p on the proliferation of CAOV3 and OVCAR3 (data are presented as mean ± SD [n = 3, each group]; *P < .05 vs pre‐NC group; ^#^ *P* < .05 vs anti‐NC group). C, Effect of miR‐542‐3p on migration and invasion of CAOV3 and OVCAR3 (data are presented as mean ± SD [n = 3, each group]; *P < .05 vs pre‐NC group; ^#^ *P* < .05 vs anti‐NC group). Photographs were taken at 200× magnification. Scale bar represents 100 μm. D, Apoptosis after CAOV3 and OVCAR3 were transfected with pre‐miR‐542‐3p and anti‐miR‐542‐3p (data are presented as mean ± SD [n = 3, each group]; **P < .01 vs pre‐NC group; ^#^ *P* < .05 vs anti‐NC group)

QRT‐PCR analysis showed that the expression level of miR‐542‐3p was significantly lower in EOC tissues than in normal ovarian tissue and was significantly lower in type II EOC tissue than in type I EOC tissue (Figure 2A). FISH analysis showed that miR‐542‐3p was also mainly expressed in the cytoplasm (Figure 1C,D), similar to circ‐PGAM1. MiR‐542‐3p‐agomir (pre‐miR‐542‐3p) and miR‐542‐3p‐antagomir (anti‐miR‐542‐3p) were separately transfected into cells to cause miR‐542‐3p overexpression and silencing, respectively. The transfection efficiency was first evaluated using qRT‐PCR (Figure S1H). CCK‐8 assay showed that the proliferation rate of cells significantly decreased in pre‐miR‐542‐3p group compared to pre‐NC group (Figure 2B). Transwell assay showed that the number of migrating and invading cells significantly decreased in pre‐miR‐542‐3p group compared to pre‐NC group (Figure 2C). Flow cytometry analysis showed that the apoptosis rate of cells significantly increased in pre‐miR‐542‐3p group compared to pre‐NC group (Figure 2D), suggesting that miR‐542‐3p overexpression could inhibit proliferation, migration, and invasion of ovarian cancer cells and promote apoptosis of ovarian cancer cells. Opposite results were obtained after miR‐542‐3p silencing. The above results indicate that miR‐542‐3p plays a tumor‐suppressing role in ovarian cancer cells.

### 3.3 MiR‐542‐3p participates in the tumor‐suppressing function mediated by circ‐PGAM1 silencing

**Figure 3:**
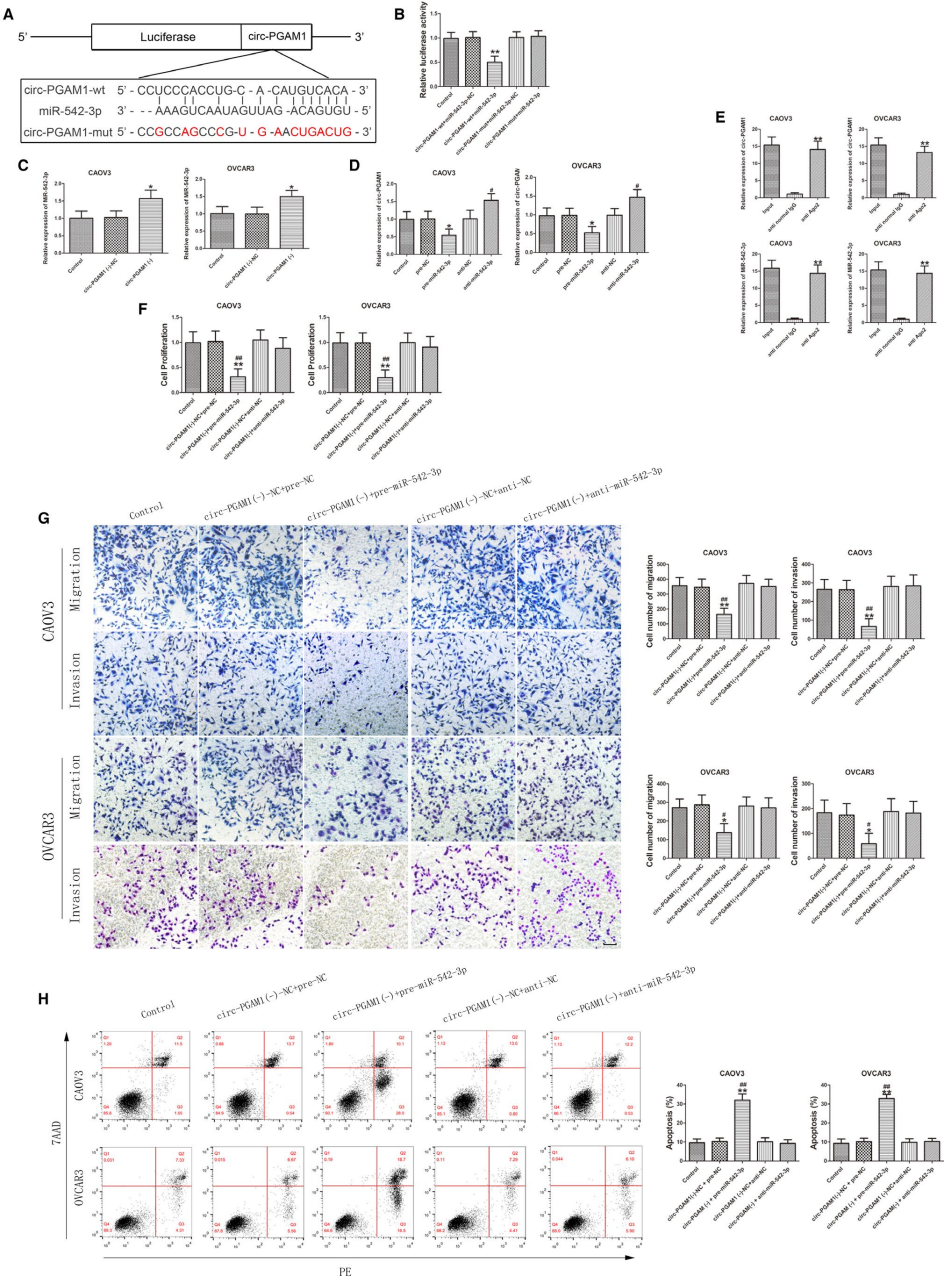
Circ‐PGAM1 promoted malignant biological behavior of epithelial ovarian cancer (EOC) cells by downregulating miR‐542‐3p expression. A, The predicted miR‐542‐3p binding site in circ‐PGAM1 (circ‐PGAM1‐Wt) and the designed mutant sequence (circ‐PGAM1‐Mut). B, Luciferase reporter assay of HEK 293T cells co‐transfected with circ‐PGAM1‐Wt or circ‐PGAM1‐Mut and miR‐542‐3p or the miR‐542‐3p‐NC (data are presented as mean ± SD [n = 3, each group]; **P < .01 vs circ‐PGAM1‐Wt + miR‐542‐3p‐NC group). C, qRT‐PCR analysis for circ‐PGAM1 regulated miR‐542‐3p expression in CAOV3 and OVCAR3 (data are presented as mean ± SD [n = 3, each group]; *P < .05 vs circ‐PGAM1 (−)‐NC group). D, qRT‐PCR for miR‐542‐3p regulated circ‐PGAM1 expression in CAOV3 and OVCAR3 (data are presented as mean ± SD [n = 3, each group]; *P < .05 vs pre‐NC group; ^#^ *P* < .05 vs anti‐NC group). E, Circ‐PGAM1 and miR‐542‐3p were identified in circ‐PGAM1‐RISC (data are presented as mean ± SD [n = 3, each group]; **P < .01 vs anti‐IgG group). F, CCK‐8 assay was performed to evaluate the effect of circ‐PGAM1 and miR‐542‐3p on the proliferation of CAOV3 and OVCAR3 (data are presented as mean ± SD [n = 3, each group]; **P < .01 vs circ‐PGAM1(−)‐NC + pre‐NC group; ^##^ *P* < .01 vs circ‐PGAM1(−)‐NC + anti‐NC group). G, Transwell assay was used to determinethe effect of circ‐PGAM1(−) and pre‐miR‐542‐3p on migration and invasion of CAOV3 and OVCAR3 (data are presented as mean ± SD [n = 3, each group]; *P < .05 vs circ‐PGAM1(−)‐NC + pre‐NC group; **P < .01 vs circ‐PGAM1(−)‐NC + pre‐NC group; ^#^ *P* < .05 vs circ‐PGAM1(−)‐NC + anti‐NC group; ^##^ *P* < .01 vs circ‐PGAM1(−)‐NC + anti‐NC group). Photographs were taken at 200× magnification. Scale bar represents 100 μm. H, Flow cytometry analysis of CAOV3 and OVCAR3 with altered expression of circ‐PGAM1 and miR‐542‐3p (data are presented as mean ± SD [n = 3, each group]; **P < .01 vs circ‐PGAM1(−)‐NC + pre‐NC group; ^##^ *P* < .01 vs circ‐PGAM1(−)‐NC + anti‐NC group)

Using a bioinformatics database (Starbase), we predicted that circ‐PGAM1, but not PGAM1 mRNA, has a potential binding site for miR‐542‐3p (Figure 3A). This interaction was first validated using a dual‐luciferase gene reporter assay. Luciferase activity was significantly lower in circ‐PGAM1‐WT (wild‐type) + pre‐miR‐542‐3p group than in circ‐PGAM1‐WT + pre‐NC group, but did not differ significantly between circ‐PGAM1‐Mut + premiR‐542‐3p group and circ‐PGAM1‐Mut + pre‐NC group (Figure 3B). Thus, these results confirmed that miR‐542‐3p binds specifically to circ‐PGAM1 at the predicted binding site. Next, qRT‐PCR analysis showed that the miR‐542‐3p expression level significantly increased in circ‐PGAM1(−) group compared to circ‐PGAM1(−)‐NC group (Figure 3C). By contrast, the circ‐PGAM1 expression level significantly decreased in pre‐miR‐542‐3p group compared to pre‐NC group (Figure 3D). RIP assay was conducted to verify whether circ‐PGAM1 andmiR‐542‐3p were involved in the RNA‐induced silencing complex (RISC). As shown in Figure 3E, expressions of circ‐PGAM1 and miR‐542‐3p were both significantly higher in anti‐Ago2 group than those in anti‐IgG group. These results indicated that miR‐542‐3p specifically targeted circ‐PGAM1in an RISC and that there might be mutual negative feedback regulation between circ‐PGAM1 and miR‐542‐3p.

To further study the effect of the joint function of circ‐PGAM1 and miR‐542‐3p on biological behaviors of ovarian cancer, cells with circ‐PGMA1 silencing combined with miR‐542‐3p silencing or overexpression were constructed. Results showed that proliferation, migration, and invasion significantly decreased in circ‐PGAM1(−) + pre‐miR‐542‐3p group compared to circ‐PGAM1(−)‐NC + pre‐NC group and circ‐PGAM1(−)‐NC + anti‐NC group (Figure 3F,G). In addition, the apoptosis rate of cells significantly increased in circ‐PGAM1(−) + pre‐miR‐542‐3p group compared to both circ‐PGAM1(−)‐NC + pre‐NC group and circ‐PGAM1(−)‐NC + anti‐NC group. However, proliferation rate of cells, migration, invasion, and apoptosis in circ‐PGAM1(−) + anti‐miR‐542‐3p group did not differ significantly from those in circ‐PGAM1(−)‐NC + pre‐NC group and circ‐PGAM1(−)‐NC + anti‐NC group (Figure 3H). The above results confirmed that circ‐PGAM1 silencing combined with miR‐542‐3p overexpression can inhibit malignant biological behaviors of ovarian cells and that miR‐542‐3p silencing could offset the tumor‐suppressing effect of circ‐PGAM1 silencing alone. These results indicate that miR‐542‐3p participates in the tumor‐suppressing function mediated by circ‐PGAM1 silencing.

### 3.4 CDC5L participates in the regulation of malignant behaviors of ovarian cancer cells mediated by circ‐PGAM1 and miR‐542‐3p

**Figure 4:**
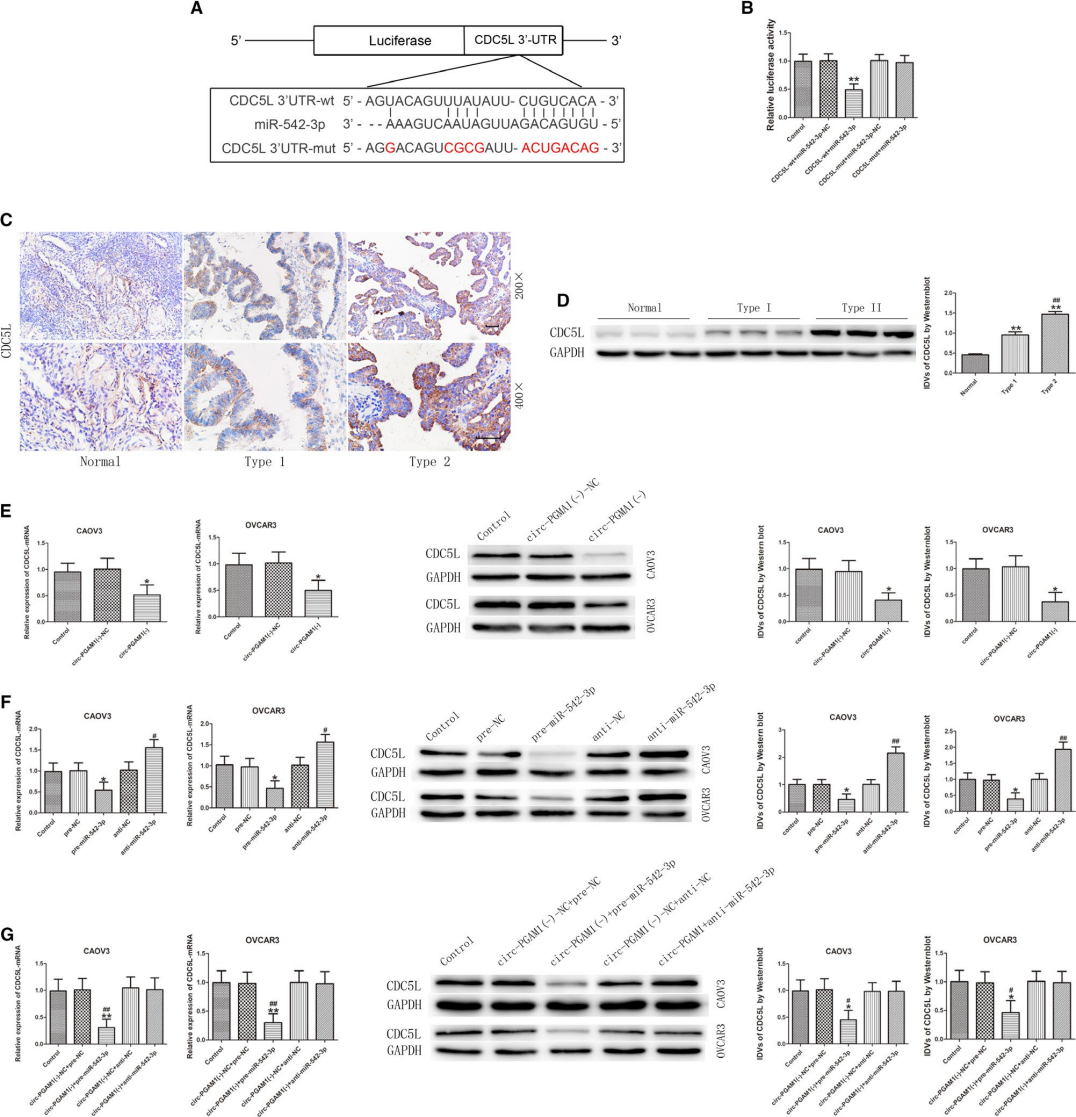
The expression of CDC5L was regulated by both circ‐PGAM1 and miR‐542‐3p in epithelial ovarian cancer (EOC). A, The predicted miR‐542‐3p binding site in CDC5L (CDC5L‐Wt) and the designed mutant sequence (CDC5L‐Mut) were indicated. B, Luciferase reporter assay of HEK 293T cells co‐transfected with CDC5L‐Wt or CDC5L‐Mut and miR‐542‐3p or miR‐542‐3p‐NC (data are presented as mean + SD [n = 3, each group]; **P < .01 vs CDC5L‐Wt + miR‐542‐3p‐NC group). C, Immunohistochemistry of CDC5L protein in normal ovarian, type I EOC, and type II EOC tissues. Original magnification: 200× and 400×. Scale bars represent 50 μm. D, CDC5L protein expression levels in normal ovarian tissues and EOC tissues of different types using GAPDH as endogenous control (data are presented as mean + SD [n = 15, each group]; **P < .01 vs normal group; ^##^ *P* < .01 vs type I EOC group). E, qRT‐PCR and western blot analysis of circ‐PGAM1 regulated CDC5L expression in CAOV3 and OVCAR3. Relative expression of CDC5L mRNA was shown using GAPDH as endogenous control. IDV of CDC5L was shown using GAPDH as endogenous control (data are presented as mean ± SD [n = 3, each group]; *P < .05 vs PGAM1 (−)‐NC group). F, qRT‐PCR and western blot analysis of miR‐542‐3p regulated CDC5L expression in CAOV3 and OVCAR3. Relative expression of CDC5L mRNA was shown using GAPDH as endogenous control. IDV of CDC5L was shown using GAPDH as endogenous control (data are presented as mean ± SD [n = 3, each group]; *P < .05 vs pre‐NC group; ^#^ *P* < .05 vs anti‐NC group; ^##^ *P* < .01 vs anti‐NC group). G, qRT‐PCR and western blot analysis of CDC5L expression regulated by circ‐PGAM1 combined with miR‐542‐3p in CAOV3 and OVCAR3. Relative expression of CDC5L mRNA was shown using GAPDH as endogenous control. IDV of CDC5L was shown using GAPDH as endogenous control (data are presented as mean ± SD [n = 3, each group]; **P < .01 vs circ‐PGAM1(−)‐NC + pre‐NC group; ^##^ *P* < .01 vs circ‐PGAM1(−)‐NC + anti‐NC group; *P < .05 vs circ‐PGAM1(−)‐NC + pre‐NC group; ^#^ *P* < .05 vs circ‐PGAM1(−)‐NC + anti‐NC group)

Using bioinformatics databases (Starbase and TargetScan), we predicted that CDC5L 3′UTR has a miR‐542‐3p binding site (Figure 4A). Next, the targeted binding between miR‐542‐3p and CDC5L was validated using a dual‐luciferase gene reporter assay. As shown in Figure 4B, luciferase activity significantly decreased in CDC5L‐Wt + pre‐miR‐542‐3p group compared to CDC5L‐Wt + pre‐NC group, but did not differ significantly between CDC5L‐Mut + pre‐miR‐542‐3p group and CDC5L‐Mut + pre‐NC group. Immunohistochemistry results showed that CDC5L protein was mainly expressed in the cytoplasm, with a low expression level in the nucleus. The expression level of CDC5L protein was significantly higher in EOC tissues than in normal ovarian tissues and was significantly higher in type II EOC than in type I EOC (Figure 4C,D). In addition, the expression levels of CDC5L protein and mRNA were detected in ovarian cells in which circ‐PGAM1 was silenced and miR‐542‐3p was silenced or overexpressed or in which circ‐PGAM1 and miR‐542‐3p were co‐transfected. The expression levels of CDC5L mRNA and protein were significantly lower in circ‐PGAM1(−) group than in circ‐PGAM1(−)‐NC group (Figure 4E), and the expression levels of CDC5L mRNA and protein were significantly lower in pre‐miR‐542‐3p group than in pre‐NC group; however, the expression levels of CDC5L mRNA and protein were significantly higher in anti‐miR‐542‐3p group than in anti‐NC group (Figure 4F). The expression levels of CDC5L mRNA and protein were significantly lower in circ‐PGAM1(−) + pre‐miR‐542‐3p group than in circ‐PGAM1(−)‐NC + pre‐NC group; however, the expression levels of CDC5L mRNA and protein did not differ significantly between circ‐PGAM1(−) + anti‐miR‐542‐3p group and the circ‐PGAM1(−)‐NC + anti‐NC group (Figure 4G). These results confirmed that the reduction of CDC5L expression caused by circ‐PGAM1 silencing is offset by miR‐542‐3p silencing and that CDC5L participates in the circ‐PGAM1/miR‐542‐3p regulatory network.

### 3.5 CDC5L plays a tumor‐promoting role in ovarian cancer cells and interacts with the promoter region of *PEAK1* gene

**Figure 5:**
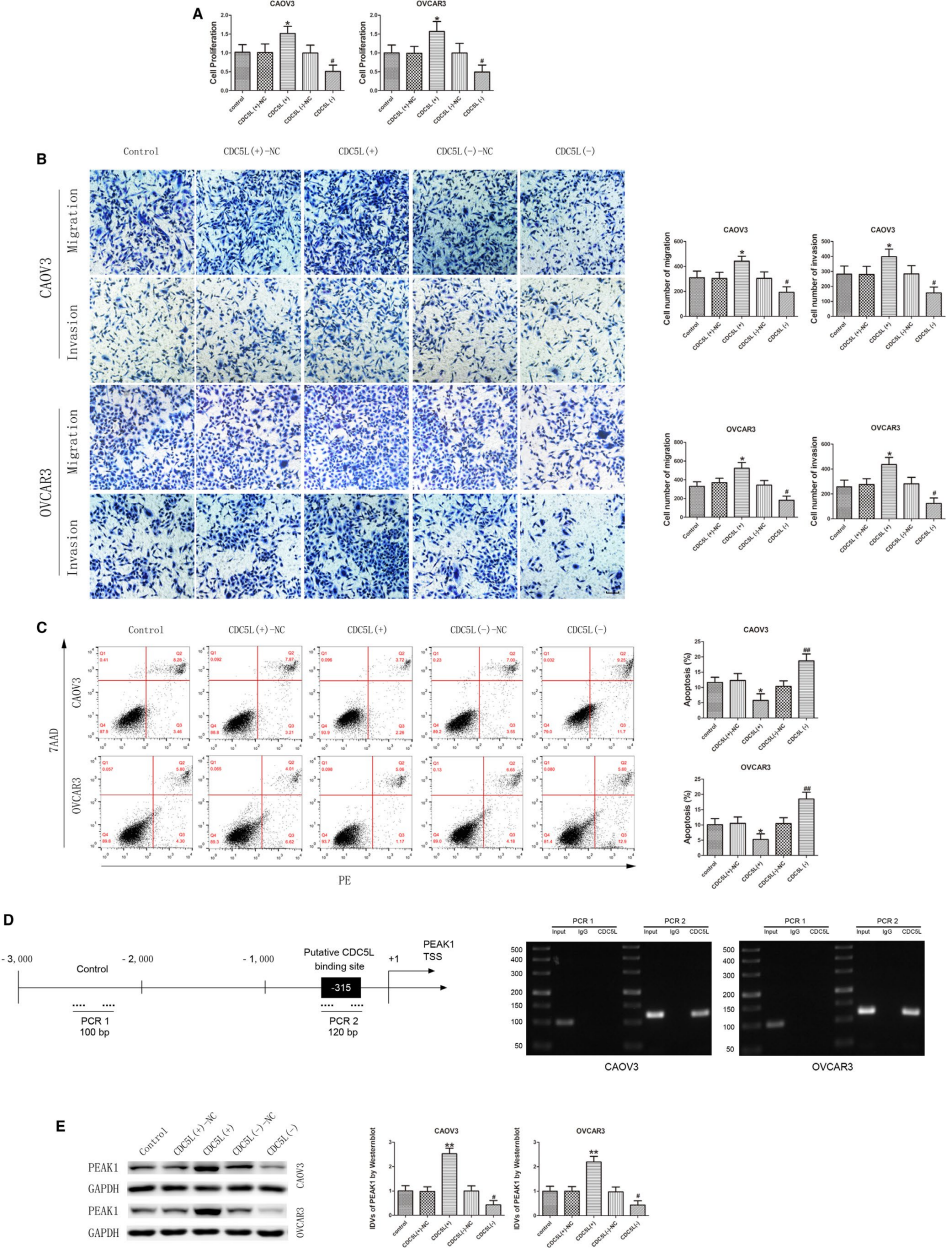
CDC5L exerted an oncogenic role and regulated PEAK1 expression in CAOV3 and OVCAR3. A, CCK‐8 assay was used to determine the effect of CDC5L on proliferation of CAOV3 and OVCAR3 (data are presented as mean ± SD [n = 3, each group]; *P < .05 vs CDC5L (+)‐NC group; ^#^ *P* < .05 vs CDC5L (−)‐NC group). B, Effect of CDC5L on migration and invasion of CAOV3 and OVCAR3 (data are presented as mean ± SD [n = 3, each group]; *P < .05 vs CDC5L (+)‐NC group; ^#^ *P* < .05 vs CDC5L (−)‐NC group). Photographs were taken at 200× magnification. Scale bar represents 100 μm. C, Flow cytometry analysis of CAOV3 and OVCAR3 with altered expression of CDC5L (data are presented as mean ± SD [n = 3, each group]; *P < .05 vs CDC5L (+)‐NC group; ^##^ *P* < .01 vs CDC5L (−)‐NC group). D, CDC5L bound to the promoter of PEAK1 in CAOV3 and OVCAR3. Transcription start site (TSS) was designated as +1. The putative CDC5L binding sites are indicated. Immunoprecipitated DNA was amplified by PCR. Normal rabbit IgG was used as negative control. E, Western blot analysis for CDC5L regulated expression of PEAK1 protein with GAPDH as endogenous control (data are presented as mean ± SD [n = 3, each group]; **P < .01 vs CDC5L (+)‐NC group; ^#^ *P* < .05 vs CDC5L (−)‐NC group)

To further study the effect of CDC5L on malignant behaviors of ovarian cancer cells, stably transfected cell lines with CDC5L overexpression or silencing were established. The transfection efficiency was evaluated by western blot (Figure S1I). After CDC5L overexpression, the proliferation rate of ovarian cancer cells and the number of migrating and invading cells both significantly increased (Figure 5A,B), whereas the apoptosis rate of the cells significantly decreased (Figure 5C). Opposite results were obtained after CDC5L silencing. The above results indicated that CDC5L plays a tumor‐promoting role in ovarian cancer cells.

Based on a bioinformatics database (JASPAR), it was predicted that CDC5L can act as a transcription factor to directly bind to the promoter region of *PEAK1* gene. To validate this prediction, chromatin immunoprecipitation (ChIP) assay was performed. The *PEAK1* promoter region sequence was confirmed using the JASPAR database. Results showed that CDC5L can directly bind to the promoter region of the *PEAK1* gene (Figure 5D). Further detection of PEAK1 protein expression in CDC5L‐overexpressing and ‐silenced ovarian cancer cell lines showed that PEAK1 protein expression level significantly increased after CDC5L overexpression, whereas PEAK1 protein expression level significantly decreased after CDC5L silencing. These results indicated that CDC5L can promote PEAK1 expression at the transcription level (Figure 5E).

### 3.6 PEAK1 promotes malignant behaviors of ovarian cancer cells through activation of the ERK1/2 and JAK2 signaling pathways

**Figure 6:**
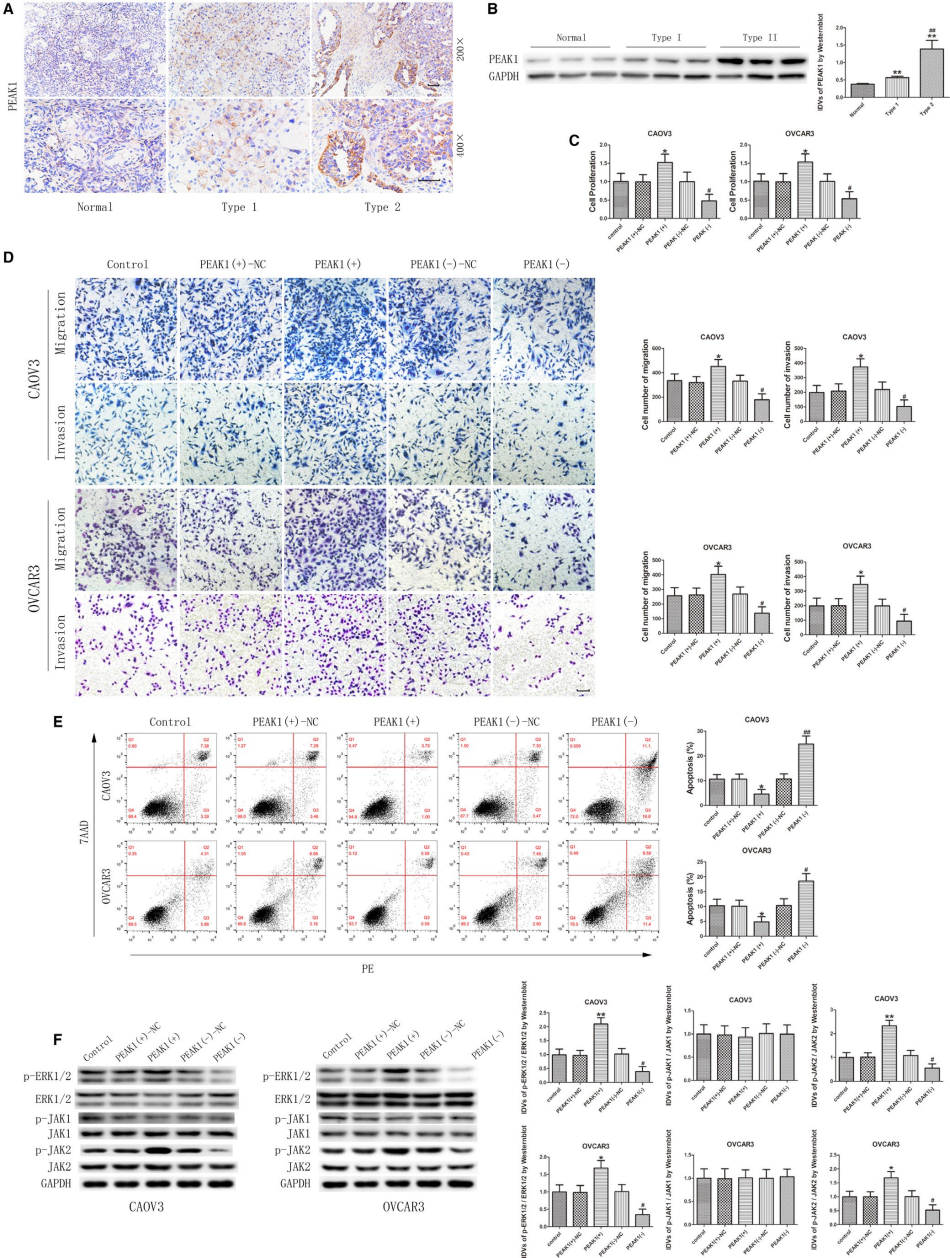
PEAK1 was upregulated in epithelial ovarian cancer (EOC) tissues and played an oncogenic role in CAOV3 and OVCAR3. A, Immunohistochemistry of PEAK1 in normal ovarian, type I EOC, and type II EOC tissues. Original magnification: 200× and 400×. Scale bars represent 50 μm. B, PEAK1 protein expression in normal ovarian tissues and EOC tissues of different types using GAPDH as endogenous control (data are presented as mean ± SD [n = 15, each group]; **P < .01 vs normal group; ^##^ *P* < .01 vs type I EOC group). C, CCK‐8 assay was used to measure the proliferation effect of PEAK1 in CAOV3 and OVCAR3 (data are presented as mean ± SD [n = 3, each group]; *P < .05 vs PEAK1 (+)‐NC group; ^#^ *P* < .05 vs PEAK1 (−)‐NC group). D, Effect of PEAK1 on migration and invasion of CAOV3 and OVCAR3 (data are presented as mean ± SD [n = 3, each group]; *P < .05 vs PEAK1 (+)‐NC group; ^#^ *P* < .05 vs PEAK1 (−)‐NC group). Photographs were taken at 200× magnification. Scale bar represents 100 μm. E, Flow cytometry analysis of CAOV3 and OVCAR3 with altered expression of PEAK1 (data are presented as mean ± SD [n = 3, each group]; *P < .05 vs PEAK1 (+)‐NC group; ^#^ *P* < .05 vs PEAK1 (−)‐NC group; ^##^ *P* < .01 vs PEAK1 (−)‐NC group). F, Western blot analysis of p‐ERK1/2, ERK1/2, p‐JAK1, JAK1, p‐JAK2, and JAK2 regulated by PEAK1 in CAOV3 and OVCAR3 with GAPDH as endogenous control (data are presented as mean ± SD [n = 3, each group]; *P < .05 vs PEAK1 (+)‐NC group; **P < .01 vs PEAK1 (+)‐NC group; ^#^ *P* < .05 vs PEAK1 (−)‐NC group)

The above results confirmed that CDC5L can upregulate PEAK1 expression. To clarify the expression and function of PEAK1 in ovarian cancer, PEAK1 expression in EOC tissues was detected using immunohistochemistry and western blot. As shown in Figure 6A,B, PEAK1 protein was mainly expressed in the cytoplasm, and its expression level was significantly higher in EOC tissues than in normal ovarian tissue and was significantly higher in type II EOC than type I EOC. Cell lines in which PEAK1 was stably overexpressed or silenced were established by transfection. The transfection efficiency was evaluated by western blot (Figure S1J). After PEAK1 overexpression, the proliferation rate of ovarian cancer cells and the number of migrating and invading cells both significantly increased (Figure 6C,D), and the apoptosis rate of cells significantly decreased (Figure 6E). PEAK1 silencing yielded opposite results. The above results indicate that PEAK1 plays a tumor‐promoting role in ovarian cancer. To further study the underlying mechanism, the activities of extracellular signal‐regulated protein kinases 1 and 2 (ERK1/2) and Janus kinase (JAK) signaling pathways were detected using western blot. As shown in Figure 6F, after PEAK1 overexpression, the expression levels of p‐ERK1/2 and p‐JAK2 significantly increased, but p‐JAK1 expression did not change significantly. These results indicate that PEAK1 can activate the ERK1/2 and JAK2 signaling pathways to further promote the malignant biological behaviors of ovarian cancer cells.

### 3.7 MiR‐542‐3p overexpression inhibits the tumor‐promotion function of CDC5L

**Figure 7:**
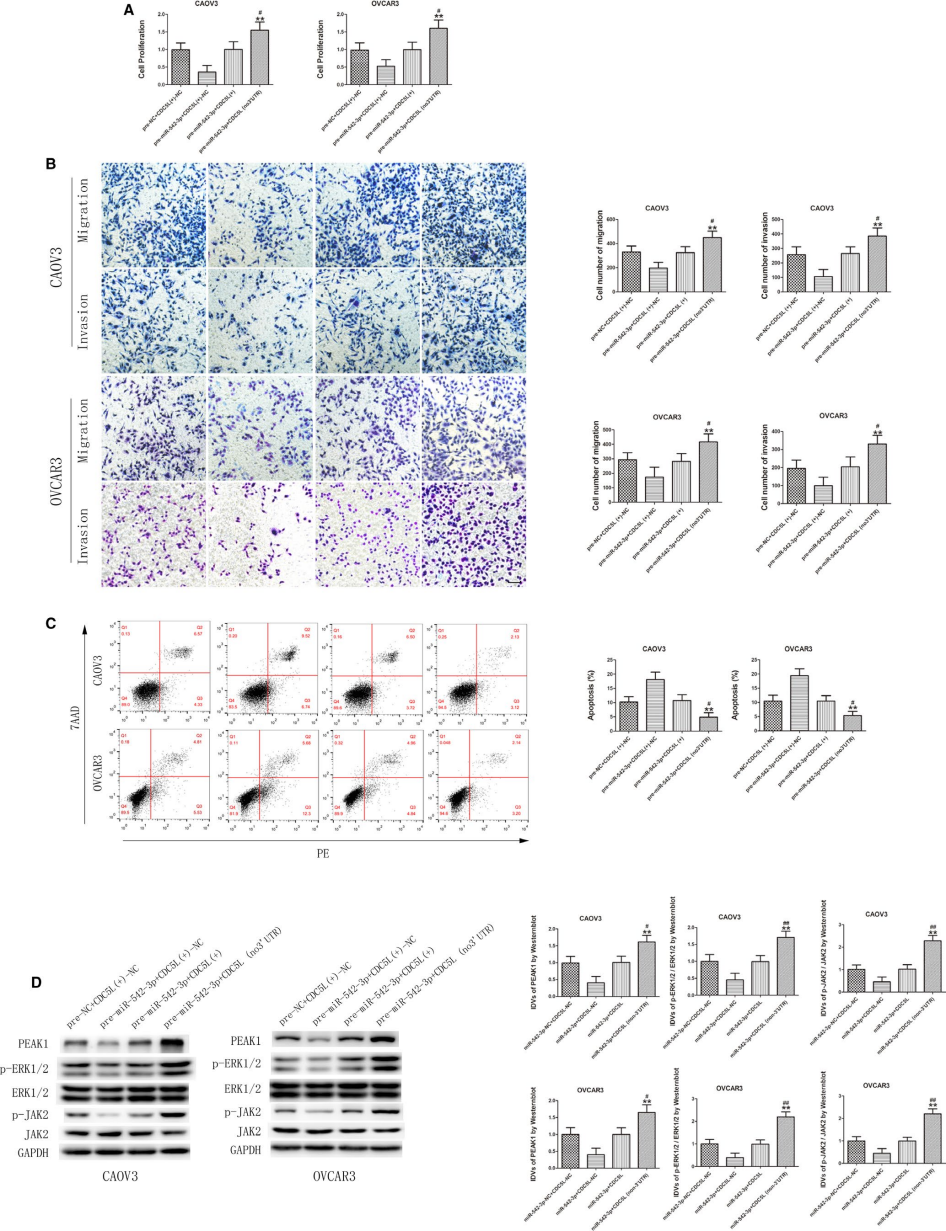
MiR‐542‐3p inhibited malignant behaviors of CAOV3 and OVCAR3 by targeting CDC5L 3′‐UTR. A, CCK‐8 assay was used to determine the effect of miR‐542‐3p and CDC5L on proliferation of CAOV3 and OVCAR3 (data are presented as mean + SD [n = 3, each group]; **P < .01 vs pre‐miR‐542‐3p + CDC5L(+)‐NC group; ^#^ *P* < .05 vs pre‐miR‐542‐3p + CDC5L(+) group). B, Transwell assay was used to detect the effect of miR‐542‐3p and CDC5L on migration and invasion of CAOV3 and OVCAR3 (data are presented as mean ± SD [n = 3, each group]; **P < .01 vs pre‐miR‐542‐3p + CDC5L(+)‐NC group; ^#^ *P* < .05 vs pre‐miR‐542‐3p + CDC5L(+) group). Photographs were taken at 200× magnification. Scale bar represents 100 μm. C, Flow cytometry analysis of CAOV3 and OVCAR3 with altered expression of miR‐542‐3p and CDC5L (data are presented as mean ± SD [n = 3, each group]; **P < .01 vs pre‐miR‐542‐3p + CDC5L(+)‐NC group; ^#^ *P* < .05 vs pre‐miR‐542‐3p + CDC5L(+) group). D, Western blot analysis of PEAK1, p‐ERK1/2, ERK1/2, p‐JAK2, and JAK2 regulated by miR‐542‐3p and CDC5L in CAOV3 and OVCAR3 with GAPDH as endogenous control (data are presented as mean + SD [n = 3, each group]; **P < .01 vs pre‐miR‐542‐3p + CDC5L(+)‐NC group; ^#^ *P* < .05 vs pre‐miR‐542‐3p + CDC5L(+) group; ^##^ *P* < .01 vs pre‐miR‐542‐3p + CDC5L(+) group)

The above results confirmed that circ‐PGAM1 can regulate CDC5L expression through miR‐542‐3p and that CDC5L promotes PEAK1 expression and activates the ERK1/2 and JAK2 signaling pathways by binding to the promoter region of *PEAK1* gene. To further study whether miR‐542‐3p inhibits CDC5L expression and plays a tumor‐suppressing role through targeted binding to the 3′‐UTR of CDC5L, an miR‐542‐3p‐overexpressing plasmid and CDC5L‐overexpressing plasmid or CDC5L 3′UTR‐deficient plasmid were co‐transfected into ovarian cancer cells to investigate their effects on malignant behaviors of ovarian cancer cells. As shown in Figure 7A,B, proliferation, migration, and invasion all significantly increased in pre‐miR‐542‐3p + CDC5L (non 3′UTR) group compared to pre‐miR‐542‐3p + CDC5L(+) group, whereas apoptosis significantly decreased (Figure 7C). In addition, PEAK1 protein expression level and activities of the ERK1/2 and JAK2 signaling pathways were detected using western blot. As shown in Figure 7D, the expression levels of PEAK1, p‐ERK1/2, and p‐JAK2 significantly increased in pre‐miR‐542‐3p + CDC5L (non‐3′UTR) group compared to pre‐miR‐542‐3p + CDC5L(+) group. These results confirmed that miR‐542‐3p regulates downstream signaling pathways through inhibition of CDC5L expression.

### 3.8 Circ‐PGAM1 silencing combined with miR‐542‐3p overexpression yielded the maximum tumor‐suppression effect in nude mice

**Figure 8:**
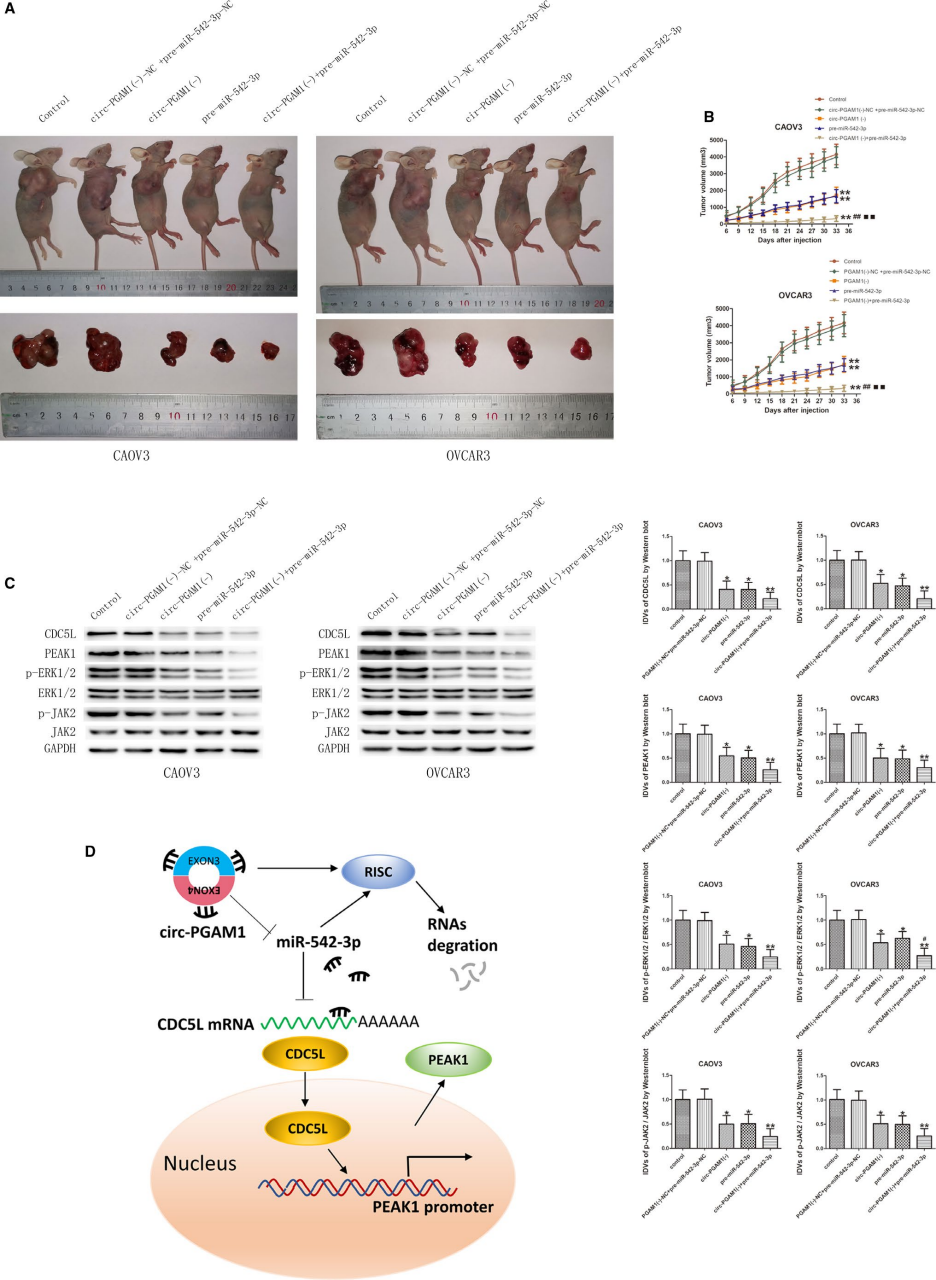
In vivo study of tumor growth and expression of relative molecules in nude mice. A, The nude mice carrying tumors with altered expression of circ‐PGAM1 and mir‐542‐3p and the sample tumors from respective groups were shown. B, The tumor size was measured every 3 d and the tumor was resected 33 d a after injection (data are presented as mean + SD [n = 3, each group]; **P < .01 vs circ‐PGAM1 (−)‐NC + pre‐miR‐542‐3p‐NC group; ^##^ *P* < .01 vs circ‐PGAM1 (−) group; ^▪▪^ *P* < .01 vs pre‐miR‐542‐3p group). C, Westernblot was used to detect the expression of p‐ERK1/2, ERK1/2, p‐JAK2 and JAK2 in tumor samples of respective groups (data are presented as mean ± SD; *P < .05 vs circ‐PGAM1 (−)‐NC + pre‐miR‐542‐3p‐NC group; **P < .01 vs circ‐PGAM1 (−)‐NC + pre‐miR‐542‐3p‐NC group; ^#^ *P* < .05 vs pre‐miR‐542‐3p group). D, Schematic cartoon of the mechanism of circ‐PGAM1 as an oncogene by regulating miR‐542‐3p/CDC5L/PEAK1 pathway in ovarian cancer cells

Based the results of the in vitro experiments above, the effects of circ‐PGAM1 and miR‐542‐3p on biological behaviors of ovarian cancer in vivo were further validated. Subcutaneous xenograft tumor experiment was performed in nude mice. As shown in Figure 8A,B, compared to circ‐PGAM1(−)‐NC + pre‐NC group, the tumor volumes significantly decreased in circ‐PGAM1(−) group, pre‐miR‐542‐3p group, and circ‐PGAM1(−) + pre‐miR‐542‐3p group. The tumor volume was lowest in circ‐PGAM1(−) + pre‐miR‐542‐3p group. These results confirmed that both circ‐PGAM1 silencing and miR‐542‐3p overexpression in nude mice can suppress ovarian cancer cell growth. In addition, circ‐PGAM1 silencing combined with miR‐542‐3p overexpression yielded the maximum tumor‐suppression effect. Further detection of the expression levels of CDC5L and PEAK1 proteins and the activities of the ERK1/2 and JAK2 signaling pathways in xenograft tumor tissues using western blot showed that the expression levels of CDC5L, PEAK1, p‐ERK1/2, and p‐JAK2 were lowest in the circ‐PGAM1 silencing combined with miR‐542‐3p overexpression group (Figure 8C).

## 4 DISCUSSION

This study confirmed that circ‐PGAM1 expression was upregulated in EOC tissues and that circ‐PGAM1 silencing inhibited the proliferation, migration, and invasion and promotes the apoptosis of ovarian cancer cells. By contrast, miR‐542‐3p expression was downregulated in EOC tissues, and miR‐542‐3p overexpression inhibited the malignant progression of ovarian cancer cells. In addition, miR‐542‐3p interacted with circ‐PGAM1 through specific sequences, and there was mutual negative feedback between circ‐PGAM1 and miR‐542‐3p. MiR‐542‐3p bound specifically to the 3′‐UTR of CDC5L to downregulate CDC5L expression. By increasing miR‐542‐3p expression, circ‐PGAM1 silencing increased its inhibitory effect on CDC5L to decrease CDC5L expression.CDC5L expression was upregulated in EOC tissues, and CDC5L overexpression promoted the malignant biological behaviors of ovarian cancer cells. In addition, the CDC5L protein directly bound to the PEAK1 promoter to promote its transcription. Furthermore, PEAK1 played a tumor‐promoting role in ovarian cancer cells. PEAK1 overexpression activated the ERK1/2 and JAK2 signaling pathways to promote malignant progression of ovarian cancer. Notably, circ‐PAGM1 silencing combined with miR‐542‐3p overexpression maximally inhibited tumor growth in in vivo studies.

With the development of bioinformatics and high‐throughput sequencing technologies in recent years, numerous circRNAs have been detected in various biological samples, and circRNAs have become a research hotspot in the noncoding RNA field.^38^ Accumulating evidence indicates that circRNAs are aberrantly expressed in various types of tumors and play important regulatory roles in tumor development and progression.^39^ Aberrantly expressed circRNAs have also been discovered in ovarian cancer. Many studies^40^, ^41^, ^42^ have confirmed that the circRNA Itchy E3 ubiquitin protein ligase (circ‐ITCH) has low expression in ovarian cancer and can inhibit proliferation, migration, and invasion and promotes apoptosis of ovarian cancer cells to exert tumor‐suppression functions. Liu et al^43^ showed that circ‐homeodomain‐interacting protein kinase (HIPK)3 (HIPK3) was highly expressed in ovarian cancer tissues and was associated with poor clinical prognosis. Zhang et al^44^ showed that hsa_circ_0051240 was highly expressed in ovarian cancer tissues and that hsa_circ_0051240 silencing inhibited proliferation, migration, and invasion of ovarian cancer cells and thereby inhibitings xenograft tumor growth in vivo. These studies suggest that circRNAs might exert oncogene or tumor‐suppressor gene functions in ovarian cancer.

As mentioned above, circ‐PGAM1 has not been studied in any type of tumor. However, its linear splicing variant, PGAM1, has been confirmed to influence aerobic glycolysis in tumor cells to exert a tumor‐promotion function in some tumors. Sun et al^17^ reported that, as a downstream molecule of the mammalian target of rapamycin (mTOR) signal transduction pathway, PGAM1 is activated by mTOR‐regulated hypoxia‐inducible factor 1α (HIF‐1α) and can promote the activity of mTOR oncogene. PGAM1 is highly expressed in nonsmall cell lung cancer tissues and is associated with poor prognosis. PGAM1 silencing inhibits mTOR‐dependent glycolysis, cell proliferation, and tumor formation. Liu et al reported that PGAM1 promoted epithelial–mesenchymal transition (EMT) of pancreatic ductal adenocarcinoma cells through activation of the Wnt/β signaling pathway and acted as a downstream target of PI3K/Akt/mTOR to promote malignant progression of pancreatic ductal adenocarcinoma.^18^ Furthermore, Wen et al^45^ reported that PGAM1 was highly expressed in prostate cancer tissues and cell lines and that PGAM1 silencing inhibited cell proliferation, migration, and migration and promoted cell apoptosis. However, this study did not detect differential expression of linear PGAM1 between EOC tissues and normal ovarian tissues. Circ‐PGAM1 expression was significantly higher in EOC tissues, and circ‐PGAM1 silencing inhibited proliferation, migration, and invasion and promoted apoptosis of ovarian cancer cells. These results indicate that PGAM1 and circ‐PGAM1 are two independent RNAs in ovarian cancer and have different biological functions. Circ‐PGAM1 exerts tumor‐promotion functions in ovarian cancer, similar to the functions of circ‐Tau tubulin kinase 2 (TTBK2), circ‐HIPK3, and circ‐SH3KBP1‐binding protein 1 (SHKBP1).^46^, ^47^, ^48^

MiR‐542‐3p expression has been reported to be downregulated in many malignant tumors such as liver cancer, colorectal cancer, and lung cancer and plays a tumor‐suppressing role. Yuan et al^49^ showed that miR‐542‐3p expression was downregulated in colorectal cancer tissues and cells and that miR‐542‐3p overexpression inhibited cell proliferation, migration, and invasion and promoted cell apoptosis through targeted binding to OTUB1. Lyu et al^24^ showed that in breast cancer, miR‐542‐3p bound specifically to three binding sites on the 3′UTR of Survivin mRNA to inhibit Survivin expression, enhance the pro‐apoptosis effect of paclitaxel in HER2‐overexpressing breast cancer cells, and inhibit HER3‐mediated drug resistance to paclitaxel. Althoff et al^23^ reported that miR‐542‐3p expression was downregulated in late‐stage, high‐grade, and MYCN‐amplified neuroblastoma. Patients with high miR‐542‐3p expression had a greater chance of progression‐free survival than patients with low miR‐542‐3p expression. In addition, miR‐542‐3p overexpression inhibited proliferation and promoted apoptosis of neuroblastoma cells. The results of this study showed that miR‐542‐3p expression was low in EOC tissues and that miR‐542‐3p overexpression inhibited ovarian cancer cell growth in vivo and in vitro. These results indicate that miR‐542‐3p also exerts tumor‐suppression functions in ovarian cancer cells.

Numerous studies have confirmed that circRNAs can act as miRNA sponges to bind miRNAs and thus regulate miRNA activities and further affect miRNA target gene expression. This study used bioinformatics software (Starbase) to show that circ‐PGAM1 had a potential miR‐542‐3p binding site. FISH showed that these two RNAs were co‐expressed in the cytoplasm of ovarian cancer cells. Dual‐luciferase experiment results further confirmed that miR‐542‐3p is bound to circ‐PGAM1, indicating that circ‐PGAM1 in ovarian cancer cells acted as an miRNA sponge to compete for miR‐542‐3p binding sites through the ceRNA mechanism to regulate miR‐542‐3p activity. This study further showed that circ‐PGAM1 silencing in ovarian cancer cells increased the expression level of miR‐542‐3p, miR‐542‐3p overexpression in ovarian cancer cells reduced the expression level of circ‐PGAM1. In addition, RIP assay results showed that circ‐PGAM1 and miR‐542‐3p were present in the RISC complex. The above results suggested that miR‐542‐3p specifically targeted circ‐PGAM1 in an RISC and that there might be mutual negative feedback regulation between circ‐PGAM1 and miR‐542‐3p.

To further validate whether circ‐PGAM1 exerts a tumor‐promotion function through regulation of miR‐542‐3p expression, circ‐PGAM1 and miR‐542‐3p were co‐transfected into ovarian cancer cells. circ‐PGAM1 silencing combined with miR‐542‐3p overexpression significantly inhibited cell proliferation, migration, and invasion and promoted cell apoptosis, whereas miR‐542‐3p silencing offset the tumor‐suppression function of simple circ‐PAGM1 silencing. Xenograft tumor experiment in nude mice also showed that circ‐PGAM1 silencing combined with miR‐542‐3p overexpression resulted in the smallest tumor volume, indicating that circ‐PGAM1 exerted tumor‐promotion function through the downregulation of miR‐542‐3p.

CDC5L has been confirmed by many studies to act as a transcription factor to exert biological functions.^28^, ^29^, ^30^, ^31^ Li et al^34^ confirmed that, as a transcription factor, CDC5L in prostate cancer cells directly interacted with the long noncoding RNA nuclear‐enriched abundant transcript 1 (NEAT1) for mutual positive regulation. In addition, CDC5L bound to the ARGN promoter to promote ARGN expression and formed a NEAT1‐CDC5L‐ARGN‐positive feedback loop to synergistically exert tumor‐promotion functions. Li et al^28^ showed that CDC5L expression was high in colorectal cancer tissues and cell lines. CDC5L also bound to and activated the hTERT promoter and promoted AKT phosphorylation. CDC5L silencing inhibited the proliferation and migration of colorectal cancer cells and inhibited tumor formation in nude mice. Our study showed that CDC5L was mainly expressed in the cytoplasm, with a low level of expression in the nucleus, suggesting that CDC5L might be a potential cytoplasmic transcription factor. After stimulation, CDC5L was activated and transferred from the cytoplasm to the nucleus to interact with gene promoters and regulate transcription. This result is consistent with the study by Bernstein et al.^31^ After the cultured cells were stimulated by serum, CDC5L rapidly translocated from the cytoplasm to the nucleus, with a concamitant increase in phosphorylation.^31^ Considering the tumor‐promotion function of CDC5L in various types of tumors and the presence of the miR‐542‐3p binding site predicted by bioinformatics database, we hypothesized that CDC5L might be involved in the regulatory network of circ‐PGAM1/miR‐542‐3p. The result of dual‐luciferase assay confirmed that CDC5L was a target gene of miR‐542‐3p. This study also confirmed that CDC5L expression was high in EOC tissues, promoted proliferation, migration, and invasion of ovarian cancer cells, and inhibited the apoptosis of ovarian cancer cells. Next, we further studied whether CDC5L was involved in circ‐PGAM1‐mediated regulation of ovarian cancer cells. As expected, circ‐PGAM1 silencing reduced CDC5L expression. Results further showed that miR‐542‐3p interacted with the CDC5L 3′UTR to inhibit CDC5L expression. Furthermore, miR‐542‐3p silencing reversed the reduction of CDC5L expression resulting from circ‐PGAM1 silencing, indicating that CDC5L participated in the regulatory network of circ‐PGAM1/miR‐542‐3p.

As mentioned above, CDC5L can promote target gene transcription. PEAK1 plays a tumor‐promoting role in many tumors. Analysis of the PEAK1 promoter region indicated a putative CDC5L binding site. ChIP confirmed our hypothesis that CDC5L can directly bind to the PEAK1 promoter. We further showed that CDC5L promoted PEAK1 expression in ovarian cancer cells, indicating that CDC5L can act as a transcription factor to activate PEAK1 expression.

PEAK1 is extensively recognized as an oncogene. Wang et al^35^ first reported that, through src‐induced tyrosine phosphorylation, PEAK1 regulated the p130cask—crk—paxillin and ERK signaling pathways and downstream integrin and epidermal growth factor receptor (EGFR) pathways to control cell spread, migration, and proliferation. Their study also showed that PEAK1 overexpression could promote the proliferation of MDA‐MB‐435 human breast cancer cells, enhance the activity of ERK kinase, and promote tumor formation in nude mice. After PEAK1 silencing in human pancreatic cancer cells, tumor growth in nude mice was suppressed. Huang et al^37^ also showed that PEAK1 expression was high in colorectal cancer tissues and that PEAK1 silencing could inhibit proliferation, migration, and invasion of colorectal cancer cells and significantly attenuate EGF‐induced p‐ERK1/2 expression levels. Ding et al^36^ showed that PEAK1 activated the ERK1/2 and JAK2 signaling pathways and promoted the malignant biological behaviors of lung cancer cells. Our study also confirmed that PEAK1 expression was high in ovarian cancer tissues and was mainly distributed in the cytoplasm. PEAK1 overexpression promoted proliferation, migration, and invasion and inhibited apoptosis of ovarian cancer cells. Further studies showed that PEAK1 overexpression increased the expression levels of p‐ERK1/2 and p‐JAK2 but did not change p‐JAK1 expression. These results indicate that PEAK1 promotes the malignant biological behaviors of ovarian cancer cells possibly or at least partially through activation of the ERK1/2 and JAK2 signaling pathways.

Since PEAK1 was activated by CDC5L and miR‐542‐3p regulated the malignant biological behaviors of ovarian cancer cells through targeting the CDC5L 3′UTR, we next examined whether PEAK1 and downstream pathways were involved in the tumor‐suppression effect of miR‐542‐3p on ovarian cancer cells. Our results showed that CDC5L (without 3′‐UTR) overexpression reversed the inhibition of malignant behaviors of ovarian cancer cells by miR‐542‐3p and increased the expression levels of PEAK1, p‐ERK1/2, and p‐JAK2. These data provided further basis for elucidation of the molecular mechanisms underlying the promotion of the development and progression of ovarian cancer by circ‐PGAM1 through regulation of the miR‐542‐3p/CDC5L/PEAK1 pathway. A schematic diagram of the specific mechanism is shown in Figure 8D.

## 5 CONCLUSIONS

In summary, our study confirmed that circ‐PGAM1 promoted proliferation, migration, and invasion and inhibited apoptosis of ovarian cancer cells through downregulation of miR‐542‐3p. MiR‐542‐3p played a tumor‐suppressing role through downregulation of CDC5L. CDC5L was a downstream target of miR‐542‐3p, and PEAK1 is a downstream target of CDC5L. This is the first study to unravel the interaction among circ‐PAGM1, miR‐542‐3p, CDC5L, and PEAK1. Furthermore, the circ‐PGAM1/miR‐542‐3p/CDC5L/PEAK1 axis might be a potential therapeutic target in ovarian cancer.

## CONFLICT OF INTEREST

None declared.

## AUTHOR CONTRIBUTION

CZ and QY designed study. CZ and YL performed the experiments. WZ and GL provided the clinical specimens. CZ analyzed the data and wrote the manuscript. All the authors read and approved the final manuscript.

## ETHICS APPROVAL AND CONSENT TO PARTICIPATE

All the parts in this study involving human participants were approved by the ethics committee of the authors' institution and were in accordance with the 1964 Helsinki declaration and its later amendments.

## CONSENT FOR PUBLICATION

Written informed consent was obtained from the patients or their authorized family for publication of this study and any accompanying images.

## Data Availability

The data that support the findings of this study are available from the corresponding author upon reasonable request.
