# Supplementary figures and images for: Circ‐PGAM1 promotes malignant progression of epithelial ovarian cancer through regulation of the miR‐542‐3p/CDC5L/PEAK1 pathway
Source: Cancer Med. 2020 Mar 13;9(10):3500–21. doi: 10.1002/cam4.2929 (PMC7221433; doi:10.1002/cam4.2929)

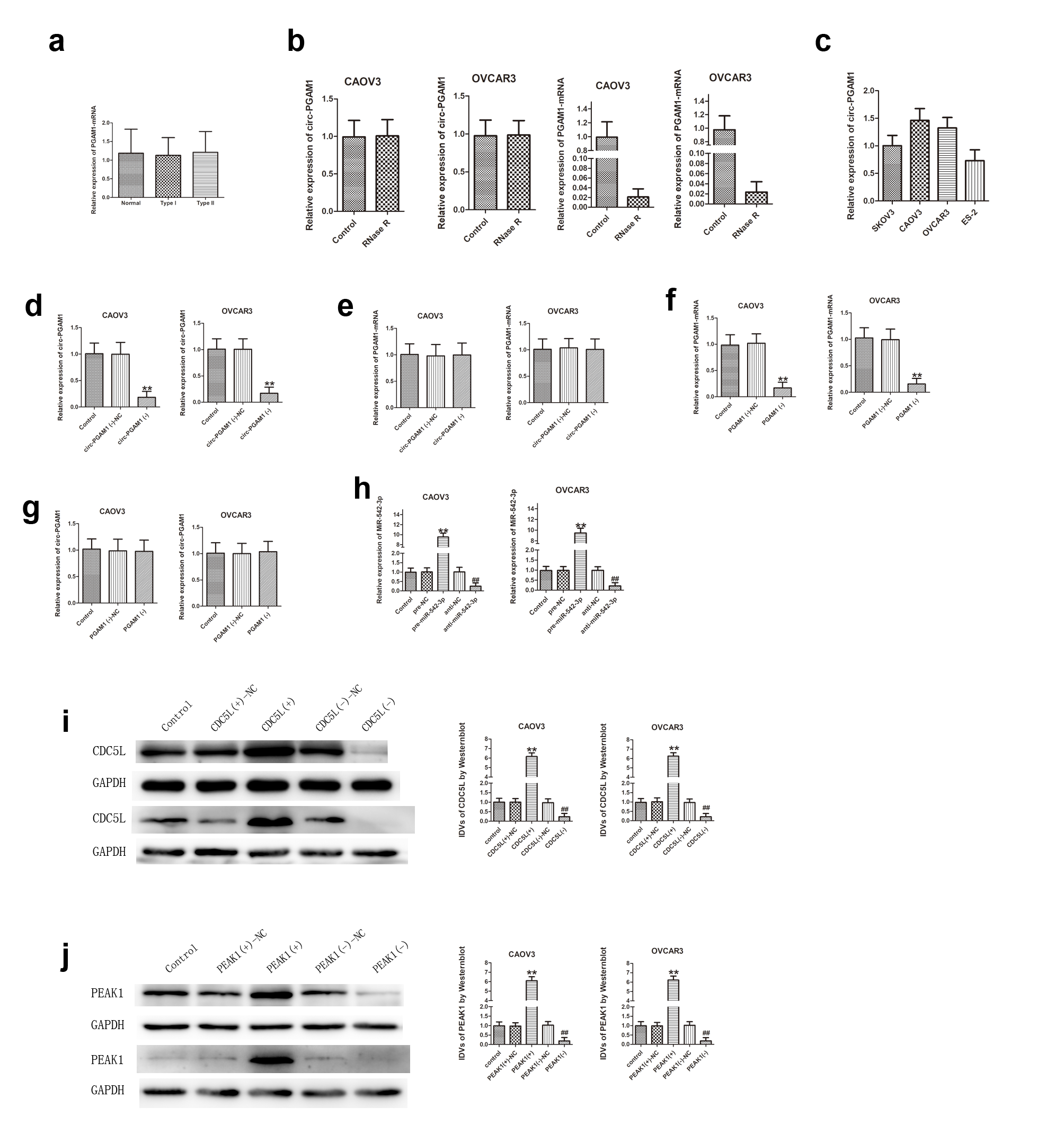

Supplement: Supplementary file 1 [file CAM4-9-3500-s001.tif]
